# A 3D Silverton-Type Polyoxomolybdate Based on {PrMo_12_O_42_}: Synthesis, Structure, Photoluminescence and Magnetic Properties

**DOI:** 10.3389/fchem.2021.615595

**Published:** 2021-02-19

**Authors:** Yanxin Zhao, Xiaopeng Sun, Yanfang Ji, Hui Kong, Shumin Chen, Pengtao Ma, Jingyang Niu, Jingping Wang

**Affiliations:** Henan Key Laboratory of Polyoxometalate Chemistry, Institute of Molecular and Crystal Engineering, College of Chemistry and Chemical Engineering, Henan University, Kaifeng, China

**Keywords:** polyoxomolybdate, crystal structure, photoluminescence, magnetic property, silverton-type

## Abstract

A three-dimensional (3D) Silverton-type polyoxomolybdate (POMo) with the formula of NH_4_{Mn_4_[PrMo_12_O_42_]}·18H_2_O (1) was successfully isolated and well characterized by single crystal X-ray diffraction, X-ray powder diffraction pattern, infrared spectrum, thermogravimetric and elemental analyses. The inorganic building block {PrMo_12_O_42_} has formed 3D frameworks *via* the {MnO_6_} linker. The excitation of compound 1 in solid state at 375 nm displays red emission. Moreover, variable temperature magnetic susceptibility measurements indicate that the magnetic behavior in compound 1 is dominated by antiferromagnetic interactions.

## Introduction

Polyoxomolybdates (POMos) represent a class of metal-oxygen clusters with remarkable structural diversity (Long et al., 2003), which have attracted much attention because of their wide ranging applications (Pope and Müller, 1991; Hill, 1998; Long et al., 2010). The inorganic building units of POMos are generally formed by the flexible Mo−O−Mo and Mo=O bonds, featuring variable coordination numbers and tunable oxidation states between Mo^V^ and Mo^VI^ (Kowalewski et al., 2012). Owing to the abundant diversity of molecular structures, examples of POMos with catalysis, magnetic (Maestre et al., 2001; Pati and Rao, 2008; Miras et al., 2011), electrochemical, and luminescent properties (T. Zhang et al., 2005; G. Zhang et al., 2017) have been sufficiently studied in the last few decades. To date, the development of POMos have mainly been focused on the classical structure-type, such as Keggin {XMo_12_O_40_} (Dolbecq et al., 2003; Yelamanchili et al., 2008; Leclerc-Laronze et al., 2009; Vasilopoulou et al., 2015) and Dawson {X_2_Mo_18_O_62_} (López et al., 2002; Sokolov et al., 2008; Hashikawa et al., 2011). For instance, Awaga et al. studied the reversible 24-electron redox during charging/discharging in [PMo_12_O_40_]^3−^ and demonstrated the potentials in making rechargeable batteries (Wang et al., 2012). On the basis of the Dawson-type anions [P_2_Mo_18_O_62_]^6−^, Poblet et al. systematically conducted the density functional theory calculations and analyzed their redox properties in detail (López et al., 2002). To the best of our knowledge, only a few of Silverton-type {XMo_12_O_42_} compounds functionalized with lanthanide (Ln) ions have been reported and the relevant research is summarized in Supplementary Table S1.

More specifically, Baker et al. reported the first Silverton-type compound in 1953 with the formula of (NH_4_)_2_H_6_[CeMo_12_O_42_]·12H_2_O and then this structure was further explored by Silverton et al. (Dexter and Silverton, 1968). Subsequently, this configuration was defined as “Silverton” type POMo, in which the [CeMo_12_O_42_]^8−^ anion is constructed by six corner-sharing {Mo_2_O_9_} groups and a twelve-coordinated Ce^4+^ ion. In 2006, Tsirlina et al. studied the electrochemical and photoluminescence properties of the aforementioned compound (Lu et al., 2006) and Gd^3+^ ion was successfully introduced in the Silverton-type POMos by the one-pot hydrothermal method (Tan et al., 2009). In addition, the transition metal nickel and cobalt ions have also been incorporated in this system, affording three isomorphic 3D frameworks (Tan et al., 2009).

Referring to the relevant systems, Ln ions maintain the inherent photoluminescence properties originated from their 4f → 4f or 5d → 4f transitions. As for the Pr^3+^ ion, the ^3^P_0_ → ^3^H_4_ transitions always emit blue-green light in most oxide lattices while the ^1^D_2_ → ^3^H_4_ transitions mainly emit red light in some pervoskite lattices (Peng et al., 2012; Liang et al., 2017). Moreover, the forbiddance of d–d transitions of the Mn^2+^ ion limits photoluminescence (Kang et al., 2018), which can be improved by the addition of photosensitizers (Lv et al., 2014) and the suitable crystal field environment (Y. Zhang et al., 2012; Cao et al., 2018).

Herein, the Pr^3+^ ion is employed to construct the Silverton-type POMo and the transition metal Mn^2+^ ion is used to extend the structural dimensionality. As expected, a new 3D Silverton-type POMo, NH_4_{Mn_4_[PrMo_12_O_42_]}·18H_2_O (**1**) has been isolated as single crystals. Under the 375 nm photoexcitation, compound **1** was detected with red emission. Furthermore, the preliminary magnetic property has also been studied by variable temperature magnetic susceptibilities measurement.

## Materials and Methods

All chemical reagents were commercially purchased from Sinopharm Chemical Reagent Co., Ltd. and used without further purification. X-ray powder diffraction data were collected on a Bruker D8 Advance diffractometer with Cu *Kα* (*λ* = 1.54056 Å) radiation. Infrared spectra were recorded on a Spectrum Two FT-IR spectrometer using KBr pellets in the range of 4,000–400 cm^−1^. Thermogravimetric analyses were carried out under N_2_ atmosphere on a Mettler Toledo TGA/DSC3 synchronous thermal analyzer (heating rate: 10°C·min^−1^). The vacuum/open 1,200°C tubular electric furnace as well as the ceramic reaction vessel are produced by Tianjin Zhonghuan Co., Ltd. Photoluminescence properties were performed at room temperature using a FLS980 fluorescence spectrophotometer. Magnetic measurements were carried out on a Quantum Design MPMS3 magnetometer in the temperature range of 310–1.8 K.

### Synthesis of Compound 1

Solid powder of Mo (0.42 g, 4.38 mmol), Na_2_MoO_4_·2H_2_O (1.06 g, 4.38 mmol) and MoCl_5_ (1.20 g, 4.39 mmol) were uniformly mixed in a 25 ml ceramic reaction vessel under the N_2_ atmosphere. Then the reaction vessel was carefully placed in the tubular electric furnace. The reactant was successively heated at 150°C (1 h), 200°C (1 h) and 240°C (2 h) and then cooled to room temperature under the protection of argon gas. Black solid powder was collected as the reaction intermediate. Subsequently, the black intermediate product (1.23 g) was dissolved in H_2_O (200 ml) and NH_3_·H_2_O (1 ml, 25–28%), and the turbid solution was stirred for 1 h, then H_2_O_2_ (24 ml, 30%) was added (pH = 4‐5). After stirred for another 3.5 h the pH of the yellow mixture was automatic adjusted to 1.9, the solution of Mn(CH_3_COO)_2_ (12 ml, 0.125 mol/L), Pr(NO_3_)_3_·4H_2_O (6 ml, 9.23 mmol/L) and H_2_O_2_ (6 ml, 30%) were added sequentially to the aforementioned mixture (30 ml). The solution was filtered after stirring for 1 h and the filtrate was left undisturbed at room temperature for crystallization. Golden-yellow block-shape crystals were obtained after about four weeks. Yield: 33.52% based on Na_2_MoO_4_·2H_2_O. Elemental analysis calcd (%) for Mn_4_Mo_12_NO_60_PrH_40_: H, 1.60; N, 0.55. Found: H, 1.65; N 0.52. The final formula of **1** should be fixed as NH_4_{Mn_4_[PrMo_12_O_42_]}·18H_2_O along with the results of thermogravimetric and elemental analyses. IR (KBr pellet, cm^−1^): 3,467 (s), 1,624 (m), 957 (m), 926 (w), 887 (w), 643 (s).

### Synthetic Discussion

The amount of hydrogen peroxide is key in the isolation of the crystals. Parallel experiments demonstrate that an insufficient amount of hydrogen peroxide leads to an undissolved molybdenum powder at the bottom of the beaker (pH > 5.1). Though the clear solution could also be obtained with excess hydrogen peroxide (pH < 3.9), a mass of flocs is separated in this clear solution in the crystallizable process. However, no single crystals were formed in the parallel experiments as described above.

### X-ray Crystallography

Single crystal X-ray structure analyses were performed on a Bruker Apex-II CCD diffractometer with graphite-monochromated Mo *Kα* radiation (*λ* = 0.71073 Å) at 296(2) K. The structure was solved by direct methods using Olex2 and the refinements were done by full-matrix least-squares against *F* ^2^. The absorption correction was performed with the SADABS program and all the Mo, Pr, Mn, and O atoms in compound **1** were refined anisotropically. The CCDC reference number is 1904951 for **1**. Crystallographic data and the structural refinement results are summarized in Table 1. The selected bond lengths and angles are listed in Supplementary Table S2.

**TABLE 1:** Crystallographic data and refinement parameters for **1**.

Compound	1
Empirical formula	Mn_4_Mo_12_NO_60_PrH_40_
Formula weight	2226.23
Temperature/K	296.15
Crystal system	Cubic
Space group	*Fd*-3
a/Å	25.9227(10)
b/Å	25.9227(10)
c/Å	25.9227(10)
Volume/Å^3^	17,420(2)
Z	2
ρ_calc_g/cm^3^	1.710
μ/mm^−1^	2.551
F(000)	8264.0
Crystal size/mm^3^	0.28 × 0.24 × 0.18
Radiation	MoKα (λ = 0.71073)
2Θ range for data collection/°	4.444 to 50.13
Index ranges	-29 ≤ h ≤ 30, -25 ≤ k ≤ 30, -18 ≤ l ≤ 30
Reflections collected	21,901
Independent reflections	1,300 [R_int_ = 0.0239, R_sigma_ = 0.0089]
Data/restraints/parameters	1,300/0/54
Goodness-of-fit on F^2^	1.190
Final R indexes [I>=2σ (I)]	R_1_ = 0.0193, wR_2_ = 0.0630
Final R indexes [all data]	R_1_ = 0.0211, wR_2_ = 0.0643
Largest diff. peak/hole/e Å^−3^	0.43/-0.34

^a^ R_1_ = Σ||F_o_|–|F_c_||/Σ|F_o_|.

^b^ wR_2_ = {Σ[w(F_o_^2^–F_c_^2^)^2^]/Σ[w(F_o_^2^)^2^]}^1/2^”

## Results and Discussion

### Structure Discussion

**FIGURE 1:**
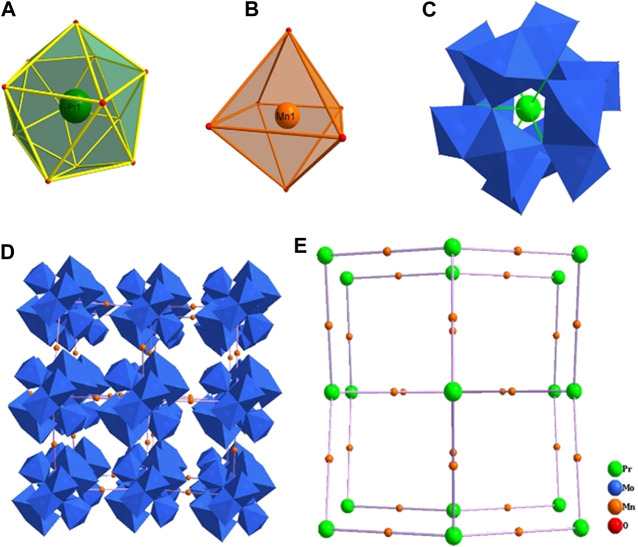
The coordination environment of Pr **(A)** and Mn **(B)** ions, the structure of the polyanion [PrMo_12_O_42_]^9−^ **(C)**, the polyhedral/ball-stick representation **(D)** and topology configuration **(E)** of the 3D framework of compound **1**.

Crystallographic analyses indicate that compound **1** is crystallized in the cubic space group *Fd*-3 and consists of one ammonium cation, four manganese cations, a Silverton-type polyanion [PrMo_12_O_42_]^9−^ and eighteen lattice water molecules. Bond valence sum (BVS) calculations reveal that Mo, Mn and Pr atoms are in +6, +2 and +3 oxidation states, respectively (Supplementary Table S3 in the Supplementary Material). In detail, the dimeric {Mo_2_O_9_} is formed by two face-sharing {MoO_6_} octahedra (Figure 1), in which the Mo−O bond lengths and the O−Mo−O bond angles are in the range of 1.691–2.339 Å and 71–165°, respectively. Six {Mo_2_O_9_} groups are connected by *μ*-O atoms forming a unit of {Mo_12_O_42_}. The twelve-coordinated Pr ion (Supplementary Figure S7A) is located in the center of the Silverton-type polyanion [PrMo_12_O_42_]^9−^ with the Pr−O bond length equals 2.559(3) Å and the O−Pr−O bond angles residing in the range of 62–178° (Supplementary Figure S7C). These polyanions are further extensively forming a 3D framework by the distorted {MnO_6_} octahedra (Supplementary Figure S7B) with the Mn−O bond length of 2.138(4) Å (Supplementary Figure S7D). Furthermore, the topology configuration of compound **1** could be seen as a 2-connected 3D network (Supplementary Figure S7E), if considering one polyanion [PrMo_12_O_42_]^9−^ as a node. In this network, each Mn ion connects to two [PrMo_12_O_42_]^9−^ units *via* O−Mn−O bridges and these bond angles are in the range of 88–180° (Supplementary Table S2 in the Supplementary Material).

### Characterizations of IR, TG and XRPD

As shown in Supplementary Figure S1, the IR spectrum of **1** shows the skeletal vibrations in the region of 1,000–600 cm^−1^. The peaks of 957 and 926 cm^−1^ are attributed to the stretching vibration of *ν*(Mo=O) and the peaks of 887 and 643 cm^−1^ are assigned to *ν*(Mo−O−Mo). In addition, the peaks appearing at 3,467 and 1,624 cm^−1^ correspond to the stretching vibration *v*(O−H) and the bending vibration *δ*(O−H) of water molecules.

The thermogravimetric (TG) curve of compound **1** exhibits two steps of weight loss (Supplementary Figure S2). The weight loss of the first step (9.79% from 25 to 200°C) is attributed to the loss of one NH_4_^+^ and 18 crystallizable water molecules. The remaining weight loss is caused by the decomposition of the skeleton of compound **1**.

The peaks in the experimental X-ray powder diffraction (XRPD) pattern are substantially consistent with the simulated peaks, indicating the phase purity of the collected sample (Supplementary Figure S3). The difference in intensity originates from the variation in preferred orientation during the collection of experimental data for the powder sample.

### Solid State Photoluminescence Properties

**FIGURE 2:**
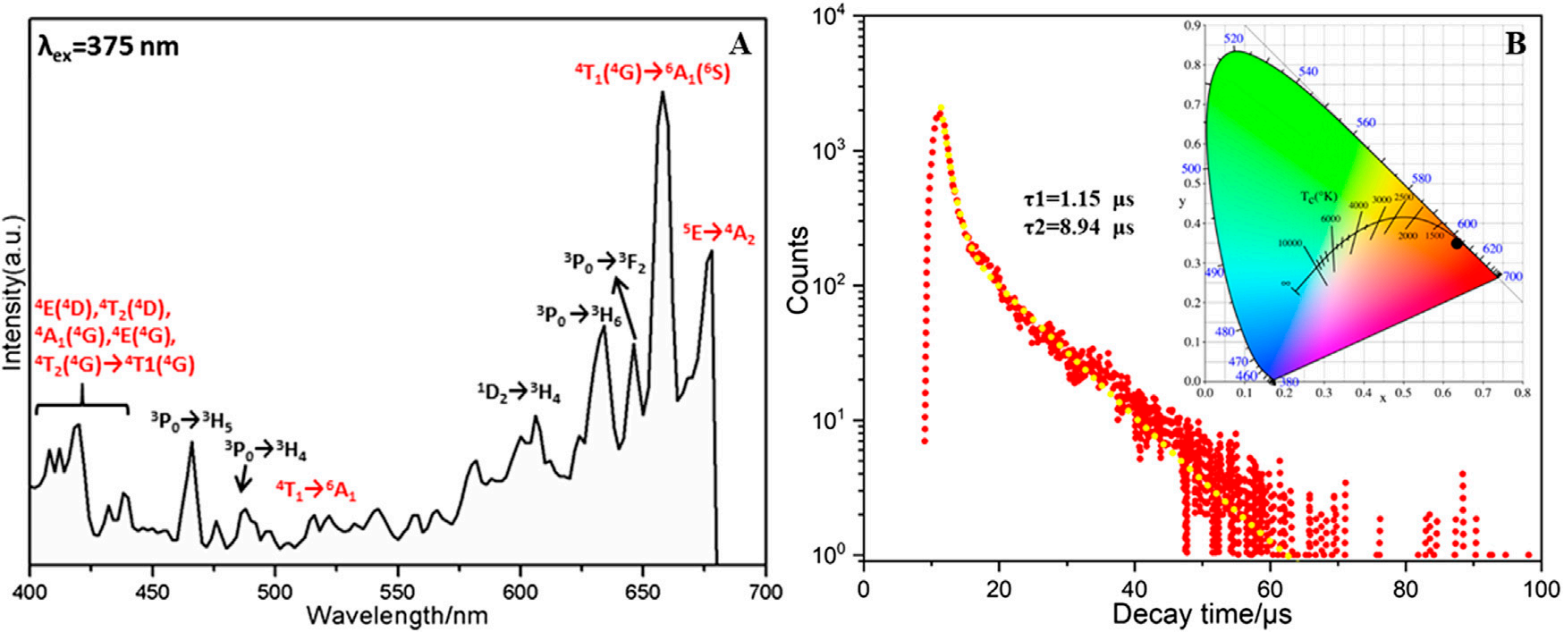
**(A)** Emission spectrum (upon 375 nm excitation) of compound **1**, **(B)** the lifetime decay curve taken by monitoring the emission at 659 nm. Inset: CIE diagram of overall emission of compound **1**.

In general, the Ln^3+^ ions possess the atomic properties in Ln-containing structures because of their gradual filling of 4f^0^ to 4f^14^ electrons in the 4f orbital and the shielding effect of 5s and 5p electrons (Ban et al., 2017). Therefore, the solid photoluminescence properties of **1** have been studied at room temperature with polycrystalline samples. Excitation of **1** in solid state at 375 nm displays two weak emission bands centered at 466 nm and 488 nm, which are individually attributed to ^3^P_0_ → ^3^H_5_ and ^3^P_0_ → ^3^H_4_ transitions of Pr^3+^ ions (Regulacio et al., 2008). The red emission bands in the range of 600–700 nm can be attributed to many factors. For example, ^1^D_2_ → ^3^H_4_ (606 nm), ^3^P_0_ → ^3^H_6_ (636 nm) and ^3^P_0_ → ^3^F_2_ (650 nm) transitions of Pr^3+^ ions (Figure 2A) (Q. Zhang et al., 2015). Besides, the strong red emission peak centered at 659 nm is assigned to the transition of Mn^2+^ ions from the higher energy levels of ^4^T_1_(^4^G) to the ground state ^6^A_1_(^6^S) (L. Wu et al., 2014), which might undergo the intermediate levels of ^4^E(^4^D), ^4^T_2_(^4^D), ^4^A_1_(^4^G), ^4^E(^4^G), and ^4^T_2_(^4^G) (Sharma et al., 2010; Shi et al., 2014). The emission band at 671 nm is attributed to the transitions from ^5^E to ^4^A_2_ of Mn^2+^ ions. These strong emission band of Mn^2+^ ions are contributed by the energy transfer from the {PrMo_12_O_42_} building block (H. Wu et al., 2018; H. Li et al., 2019). In other words, the Mo−O−Mo linker may play a role of light radiation absorption as well as the energy transfer processes (Pan et al., 2018). In addition, the red emission spectra of Mn^2+^ ions in **1** is in line with the crystal field environment, i.e., orange to deep red emission for octahedral coordination configuration (L. Wu et al., 2014). It should be noted that the intensity of the emission band for Mn^2+^ is greatly stronger than that of Pr^3+^. The photoluminescence intensity is related to the concentration of ions and the concentration of Mn^2+^ is four times that of the Pr^3+^ ion in compound **1** (Mhlongo et al., 2011; Pan et al., 2018; X. Yang et al., 2019).

Under the most intense emission at 659 nm, the characteristic excitation peaks of Pr^3+^ ion are centered at 475, 479, and 496 nm for the transition of ^3^H_4_ → ^3^P_2_, ^3^H_4_ → ^3^P_1_ and ^3^H_4_ → ^3^P_0_, respectively (Regulacio et al., 2008). The transition of ^6^A_1g_(^6^S) → ^4^T_2g_(^4^D) (Shang et al., 2015), ^6^A_1_ → ^4^E(^4^D), ^6^A_1_ → ^4^T_2_(D) and ^6^A_1_ → [^4^A_1_(G), ^4^E(^4^G)] (K. Li et al., 2016) for Mn^2+^ ion are obtained at 375, 390, 425, and 457 nm (Supplementary Figure S9).

### Decay Analyses

The decay lifetime of **1** in solid state was measured under the strongest emission band at 659 nm (Ban et al., 2017). As shown in Figure 2B, the decay curve is fitted well using the second order exponential function with lifetimes of *τ*_1_ = 1.15 μs(50.02%) and *τ*_2_ = 8.94 μs (49.98%) (H.-L. Li et al., 2017). The average decay time of 5.04 μs (τ*) can be obtained using the following equation: τ* = (*A*_1_ *τ*_1_^2^+*A*_2_ *τ*_2_^2^)/(*A*_1_ *τ*_1_+*A*_2_ *τ*_2_) (A_1_ = 2008.28, A_2_ = 257.76) (Tian et al., 2016). In addition, the chromaticity coordinates (*x*, *y*) are always used to determine the emission color of samples and the standard chromaticity coordinates of white light emission is (0.33, 0.33). For compound **1**, the chromaticity coordinates are found to be (0.641, 0.359). Therefore, the overall emission of **1** can be simulated to be red emission, which is close to the reported analogues (Geng et al., 2013).

### Magnetic Property

**FIGURE 3:**
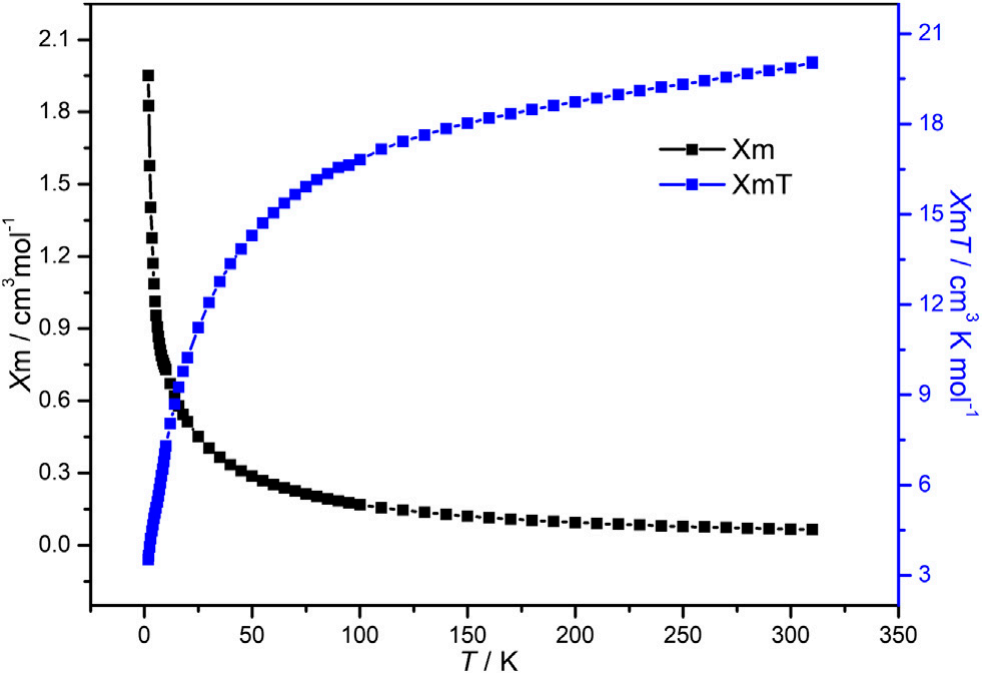
Plots of _*χ*M_ and _*χ*M_ *T* vs. *T* from 310 K to 1.8 K of compound **1**.

The variable-temperature magnetic susceptibility of compound **1** in the polycrystalline state has been investigated at 1,000 Oe at temperature ranges of 310–1.8 K. As shown in Figure 3, the experimental *χ*_M_ *T* product of 19.9 cm·K·mol^−1^ at room temperature is close to the expected value of 19.1 cm·K·mol^−1^ for one Pr^3+^ ion and six uncoupled high-spin Mn^2+^ ions (L. Yang et al., 2015; Martínez-Coronado et al., 2012; Jia et al., 2018). Upon cooling, the *χ*_M_ *T* value gradually decreases to 14.3 cm·K·mol^−1^ at 50 K, below which the *χ*_M_ *T* value sharply drops to 3.5 cm·K·mol^−1^ at 1.8 K. The reciprocal susceptibility 1/*χ*_M_ *vs. T* plots align well with the Curie-Weiss law from 300 to 20 K with a negative Weiss constant of −24.96 K (Supplementary Figure S10), which further proves that the magnetic properties of compound **1** is dominated by antiferromagnetic interactions (Nunzi et al., 2007; Zhao et al., 2019). These antiferromagnetic coupling might be transferred by O−Mo−O bonds (Castellano et al., 2011).

## Conclusion

In summary, a new 3D POMo framework imbedding Ln and transition metal ions has been synthesized and characterized. To the best of our knowledge, our work, employing the Silverton-type building block {XMo_12_O_42_}, is able to enrich the Silverton-type POMo family. The twelve-coordinated Pr^3+^ ion in compound **1** is encapsulated at the center of the Silverton-type {PrMo_12_O_42_} and the bridged Mn^2+^ ions further extend the structure to 3D frameworks. The overall red emission spectra of **1** originates from the nature of Pr^3+^ and Mn^2+^ ions and the lifetime decay behavior has also been systematically probed [*τ*_1_ = 1.15 μs (50.02%) and *τ*_2_ = 8.94 μs (49.98%)]. Magnetic property studies indicate that **1** displays antiferromagnetic interactions. The successful isolation of **1** is not only beneficial for the development of Ln-containing POMs, but also opens a way to explore multifunctional material with photoluminescence and magnetic properties.

## Data Availability

The datasets presented in this study can be found in online repositories. The names of the repository/repositories and accession number(s) can be found in the article/Supplementary Material.
