# Supplementary material for: A 3D Silverton-Type Polyoxomolybdate Based on {PrMo12O42}: Synthesis, Structure, Photoluminescence and Magnetic Properties
Source: Front Chem. 2021 Feb 19;9:615595. doi: 10.3389/fchem.2021.615595 (PMC7935544; doi:10.3389/fchem.2021.615595)
Supplement: Supplementary file 1 [file table1.docx]

Supplementary Material

Yanxin Zhao, Xiaopeng Sun, Yanfang Ji, Hui Kong, Shumin Chen, Pengtao Ma, Jingyang Niu^*^ and Jingping Wang^*^

*Henan Key Laboratory of Polyoxometalate Chemistry, Institute of Molecular and Crystal Engineering, College of Chemistry and Chemical Engineering, Henan University, Kaifeng, Henan 475004 (P. R. China)*

***Correspondence:**Jingyang Niu* and Jingping Wang*
[jyniu@henu.edu.cn](mailto:jyniu@henu.edu.cn), [jpwang@henu.edu.cn](mailto:jpwang@henu.edu.cn)

**Table S1**. A survey of relevant reports of Ln-containing Silverton-type POMos.

**Table S2.** Selected bond length (Å) and bond angle (°) of compound **1**.

**Table S3.** Bond valence sum parameters for Pr, Mo and Mn atoms in compound **1**.

**Figure S1.** The infrared spectrum of compound **1**.

**Figure S2.** Thermogravimetric analysis of compound **1**.

**Figure S3.** The X-ray powder diffraction pattern of compound **1**.

**Figure S4.** Single-crystal morphology observed by optical microscope of **1**.

**Figure S5.** The relative position of the Pr^3+^ and Mn^2+^ in compound **1**.

**Figure S6.** The coordination environment of Pr^3+^ and Mn^2+^ in compound **1**.

**Figure S7.** The representation of the assembly of compound **1**.

**Figure S8.** The space-filling view of compound **1**.

**Figure S9.** The excitation spectrum of compound **1**.

**Figure S10.** Plots of *χ*_M_ and 1/*χ*_M_ *versus* *T* of compound **1**.

**Table S1**. A survey of relevant reports of Ln-containing Silverton-type POMos.

| Formula | Ln | Ref. |
| --- | --- | --- |
| (NH_4_)_2_H_6_[CeMo_12_O_42_]·12H_2_O | Ce | Silverton *et al*.^1^ |
| [Gd(H_2_O)_3_]_3_[GdMo_12_O_42_]·3H_2_O | Gd | Lu *et al*.^2^ |
| (NH_4_)_2_H_6_[CeMo_12_O_42_]·12H_2_O | Ce | Tsirlina, *et al*.^3^ |
| [Li(H_2_O)_4_]_2_Co(H_2_O)_4_Ce(H_2_O)_3_[CeMo_12_O_42_]·3H_2_O | Ce | Wang *et al*.^4^ |
| H_0.5_[Li(H_2_O)_4_]_2.5_[Ni(H_2_O)_4_]_0.5_Ce(H_2_O)_3_[CeMo_12_O_42_]·3H_2_O | Ce | Wang *et al*.^4^ |
| H[Li(H_2_O)_4_]_3_Ce(H_2_O)_3_[CeMo_12_O_42_]·3H_2_O | Ce | Wang *et al*.^4^ |

Ref:

1. Dexter, David D., and J. V. Silverton. 1968. “A New Structural Type for Heteropoly Anions. The Crystal Structure of (NH_4_)_2_H_6_(CeMo_12_O_42_).12H_2_O.” *Journal of the American Chemical Society* 90 (13): 3589–90. https://doi.org/10.1021/ja01015a067.
2. Wu, Chuan-De, Can-Zhong Lu, Hong-Hui Zhuang, and Jin-Shun Huang. 2002. “Hydrothermal Assembly of a Novel Three-Dimensional Framework Formed by [GdMo_12_O_42_]^9-^ Anions and Nine Coordinated Gd^III^ Cations.” *Journal of the American Chemical Society* 124 (15): 3836–37. https://doi.org/10.1021/ja017782w.
3. Lu, Yu Wei, Bineta Keita, Louis Nadjo, Gérard Lagarde, Eric Simoni, Guangjin Zhang, and Galina A. Tsirlina. 2006. “Excited State Behaviors of the Dodecamolybdocerate (IV) Anion: (NH_4_)_6_H_2_ (CeMo_12_O_42_)·9H_2_O.” *The Journal of Physical Chemistry B* 110 (31): 15633–39. https://doi.org/10.1021/jp0605653.
4. Tan, Huaqiao, Weilin Chen, Yang-Guang Li, Ding Liu, Limin Chen, and Enbo Wang. 2009. “A Series of Pure Inorganic Eight-Connected Self-Catenated Network Based on Silverton-Type Polyoxometalate.” *Journal of Solid State Chemistry* 182 (3): 465–70. https://doi.org/10.1016/j.jssc.2008.11.011.

**Table S2.** Selected bond length (Å) and bond angle (°) of compound **1**.

| Bond | Length | Bond | Length | Bond | Length |
| --- | --- | --- | --- | --- | --- |
| Pr1-O1 | 2.555(2) | Pr1-O1^10^ | 2.555(2) | Mn1-O4^8^ | 2.140(2) |
| Pr1-O1^1^ | 2.555(2) | Pr1-O1^11^ | 2.555(2) | Mn1-O4 | 2.140(2) |
| Pr1-O1^2^ | 2.555(2) | Mo1-O1^7^ | 2.249(2) | Mn1-O4^14^ | 2.140(2) |
| Pr1-O1^3^ | 2.555(2) | Mo1-O1 | 1.900(2) | Mn1-O4^11^ | 2.140(2) |
| Pr1-O1^4^ | 2.555(2) | Mo1-O1^11^ | 2.339(2) | O1-Mo1^6^ | 2.249(2) |
| Pr1-O1^5^ | 2.555(2) | Mo1-O2 | 1.9467(18) | O1-Mo1^8^ | 2.339(2) |
| Pr1-O1^6^ | 2.555(2) | Mo1-O3 | 1.691(2) | O2-Mo1^5^ | 1.9467(18) |
| Pr1-O1^7^ | 2.555(2) | Mo1-O4 | 1.738(2) |  |  |
| Pr1-O1^8^ | 2.555(2) | Mn1-O4^12^ | 2.140(2) |  |  |
| Pr1-O1^9^ | 2.555(2) | Mn1-O4^13^ | 2.140(2) |  |  |
| Bond | Angle | Bond | Angle | Bond | Angle |
| O1-Pr1-O1^1^ | 116.81(3) | O1^9^-Pr1-O1^7^ | 63.61(10) | O2-Mo1-O1^9^ | 73.10(8) |
| O1^2^-Pr1-O1^3^ | 116.81(3) | O1^2^-Pr1-O1^7^ | 116.81(3) | O2-Mo1-O1^7^ | 71.04(8) |
| O1^4^-Pr1-O1^5^ | 63.61(10) | O1-Pr1-O1^2^ | 116.40(3) | O3-Mo1-Pr1 | 126.04(9) |
| O1^4^-Pr1-O1^3^ | 116.41(10) | O1^11^-Pr1-O1^7^ | 178.86(9) | O3-Mo1-Mo1^5^ | 120.84(9) |
| O1^6^-Pr1-O1^7^ | 64.27(8) | O1-Pr1-O1^5^ | 116.41(10) | O3-Mo1-O1 | 103.71(11) |
| O1^8^-Pr1-O1^2^ | 116.40(3) | O1^10^-Pr1-O1^5^ | 116.81(3) | O3-Mo1-O1^9^ | 93.77(10) |
| O1^9^-Pr1-O1^3^ | 116.40(3) | O1^10^-Pr1-O1^2^ | 63.61(10) | O3-Mo1-O1^7^ | 164.75(10) |
| O1^8^-Pr1-O1^3^ | 62.52(8) | O1^10^-Pr1-O1^8^ | 116.81(3) | O3-Mo1-O2 | 100.43(10) |
| O1^10^-Pr1-O1^7^ | 116.40(3) | O1^1^-Pr1-O1^10^ | 178.86(9) | O3-Mo1-O4 | 103.55(12) |
| O1^4^-Pr1-O1^6^ | 116.81(3) | O1^6^-Pr1-O1^5^ | 116.40(3) | O4-Mo1-Pr1 | 121.95(8) |
| O1^1^-Pr1-O1^5^ | 62.52(8) | O1^9^-Pr1-O1^2^ | 64.27(8) | O4-Mo1-Mo1^5^ | 117.44(8) |
| O1^9^-Pr1-O1^5^ | 64.27(8) | O1^2^-Pr1-O1^5^ | 64.27(8) | O4-Mo1-O1^9^ | 162.20(10) |
| O1^7^-Pr1-O1^5^ | 62.52(8) | O1^11^-Pr1-O1^5^ | 116.40(3) | O4-Mo1-O1^7^ | 90.51(10) |
| O1-Pr1-O1^11^ | 116.81(3) | O1-Pr1-O1^3^ | 63.60(10) | O4-Mo1-O1 | 100.51(10) |
| O1^8^-Pr1-O1^11^ | 63.61(10) | O1^6^-Pr1-O1^2^ | 178.86(9) | O4-Mo-O2 | 99.35(9) |
| O1^5^-Pr1-O1^3^ | 178.86(9) | O1-Pr1-O1^9^ | 62.52(8) | O4^12^-Mn1-O4^13^ | 88.84(9) |
| O1^10^-Pr1-O1^9^ | 62.52(8) | O1^1^-Pr1-O1^3^ | 116.40(3) | O4^12^-Mn1-O4 | 91.16(9) |
| O1^8^-Pr1-O1^6^ | 62.52(8) | O1^1^-Pr1-O1^11^ | 116.81(3) | O4^7^-Mn1-O4^14^ | 91.16(9) |
| O1^8^-Pr1-O1^9^ | 178.86(9) | O1^9^-Pr1-O1^4^ | 116.81(3) | O4^7^-Mn1-O4^6^ | 88.84(9) |
| O1^4^-Pr1-O1^7^ | 116.40(3) | O1^9^-Pr1-O1^11^ | 116.41(10) | O4^6^-Mn1-O4 | 88.84(9) |
| O1-Pr1-O1^4^ | 178.86(9) | O1^10^-Pr1-O1^11^ | 64.27(8) | O4^7^-Mn1-O4^13^ | 91.16(9) |
| O1^1^-Pr1-O1^2^ | 116.41(10) | O1^4^-Pr1-O1^11^ | 62.52(8) | O4^14^-Mn1-O4^6^ | 180 |
| O1^1^-Pr1-O1^4^ | 64.27(8) | O1-Pr1-O1^6^ | 64.27(8) | O4^7^-Mn1-O4 | 88.84(9) |
| O1^8^-Pr1-O1^5^ | 116.81(3) | O1^1^-Pr1-O1^6^ | 63.61(10) | O4^14^-Mn1-O4 | 91.16(9) |
| O1^10^-Pr1-O1^4^ | 116.40(3) | O1-Pr1-O1^8^ | 116.40(3) | O4^12^-Mn1-O4^14^ | 88.84(9) |
| O1^4^-Pr1-O1^2^ | 62.52(8) | O1^6^-Pr1-O1^11^ | 116.40(3) | O4^12^-Mn1-O4^6^ | 91.16(9) |
| O1^8^-Pr1-O1^4^ | 64.27(8) | O1^7^-Pr1-O1^3^ | 116.81(3) | O4^13^-Mn1-O4^6^ | 91.16(9) |
| O1^10^-Pr1-O1^3^ | 64.27(8) | Mo1^5^-Mo1-Pr1 | 63.390(4) | O4^13^-Mn1-O4^14^ | 88.84(9) |
| O1^6^-Pr1-O1^3^ | 62.52(8) | O1-Mo1-Pr1 | 44.80(7) | O4^12^-Mn1-O4^7^ | 180 |
| O1^11^-Pr1-O1^3^ | 64.27(8) | O1^7^-Mo1-Pr1 | 46.43(5) | O4^13^-Mn1-O4 | 180.00(11) |
| O1^1^-Pr1-O1^9^ | 116.40(3) | O1^9^-Mo1-Pr1 | 46.31(6) | Mo1^10^-O1-Pr1 | 94.17(7) |
| O1-Pr1-O1^10^ | 62.51(8) | O1^7^-Mo1-Mo1^5^ | 45.34(5) | Mo1^6^-O1-Pr1 | 92.02(7) |
| O1^2^-Pr1-O1^11^ | 62.52(8) | O1-Mo1-Mo1^5^ | 108.17(7) | Mo1-O1-Pr1 | 103.60(9) |
| O1^10^-Pr1-O1^6^ | 116.41(10) | O1^9^-Mo1-Mo1^5^ | 47.72(5) | Mo1-O1-Mo1^10^ | 132.60(11) |
| O1-Pr1-O1^7^ | 64.27(8) | O1-Mo1-O1^7^ | 79.02(12) | Mo1^10^-O1-Mo1^6^ | 86.94(7) |
| O1^1^-Pr1-O1^7^ | 62.52(8) | O1-Mo1-O1^9^ | 78.96(12) | Mo1-O1-Mo1^6^ | 134.79(11) |
| O1^8^-Pr1-O1^7^ | 116.41(10) | O1^9^-Mo1-O1^7^ | 71.85(9) | Mo1^5^-O2-Mo1 | 108.37(14) |
| O1^9^-Pr1-O1^6^ | 116.81(3) | O1-Mo1-O2 | 143.97(10) | Mo1-O2-Mo1^5^ | 108.2(2) |
| O1^1^-Pr1-O1^8^ | 64.27(8) | O2-Mo1-Pr1 | 99.20(7) | Mo1-O4-Mn1 | 143.4(2) |

**Table S3.** Bond valence sum parameters for Pr, Mo and Mn atoms in compound **1**.

| Bond | Bond length | Bond Valence | Valence Sum |
| --- | --- | --- | --- |
| Pr1-O1 | 2.555 | 0.263835 | ∑(Pr1) =3.166017 |
| Pr1-O1^1^ | 2.555 | 0.263835 |  |
| Pr1-O1^2^ | 2.555 | 0.263835 |  |
| Pr1-O1^3^ | 2.555 | 0.263835 |  |
| Pr1-O1^4^ | 2.555 | 0.263835 |  |
| Pr1-O1^5^ | 2.555 | 0.263835 |  |
| Pr1-O1^6^ | 2.555 | 0.263835 |  |
| Pr1-O1^7^ | 2.555 | 0.263835 |  |
| Pr1-O1^8^ | 2.555 | 0.263835 |  |
| Pr1-O1^9^ | 2.555 | 0.263835 |  |
| Pr1-O1^10^ | 2.555 | 0.263835 |  |
| Pr1-O1^11^ | 2.555 | 0.263835 |  |
| Mo1-O1^7^ | 2.249 | 0.396799 | ∑(Mo1) =5.997034 |
| Mo1-O1 | 1.9 | 1.019099 |  |
| Mo1-O1^11^ | 2.339 | 0.311123 |  |
| Mo1-O2 | 1.9467 | 0.898259 |  |
| Mo1-O3 | 1.691 | 1.792809 |  |
| Mo1-O4 | 1.738 | 1.578945 |  |
| O1-Mo1^6^ | 2.249 | 0.396799 |  |
| O1-Mo1^8^ | 2.339 | 0.311123 |  |
| O2-Mo1^5^ | 1.9467 | 0.898259 |  |
| Mn1-O4^12^ | 2.14 | 0.351359 | ∑(Mn1) =2.108156 |
| Mn1-O4^13^ | 2.14 | 0.351359 |  |
| Mn1-O4^8^ | 2.14 | 0.351359 |  |
| Mn1-O4 | 2.14 | 0.351359 |  |
| Mn1-O4^14^ | 2.14 | 0.351359 |  |
| Mn1-O4^11^ | 2.14 | 0.351359 |  |


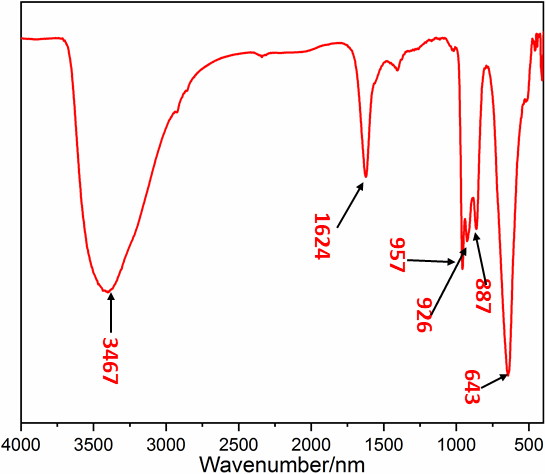


**Figure S1.** The infrared spectrum of compound **1**.


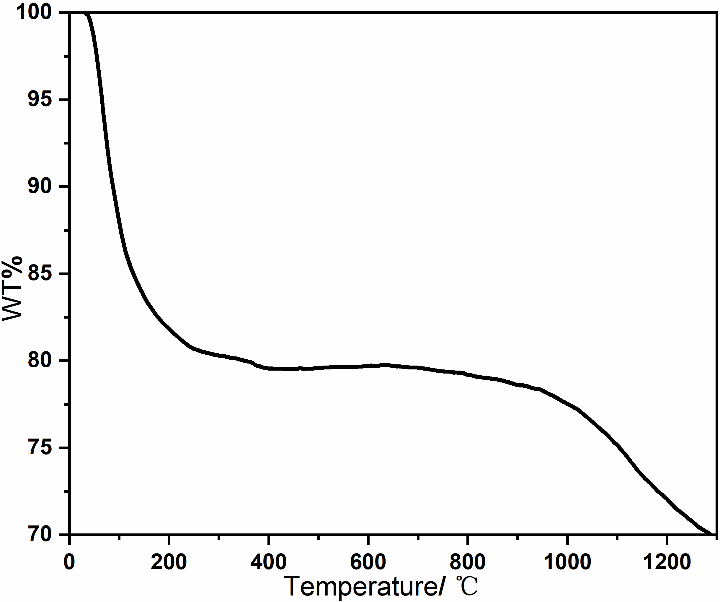


**Figure S2.** Thermogravimetric analysis of compound **1**.


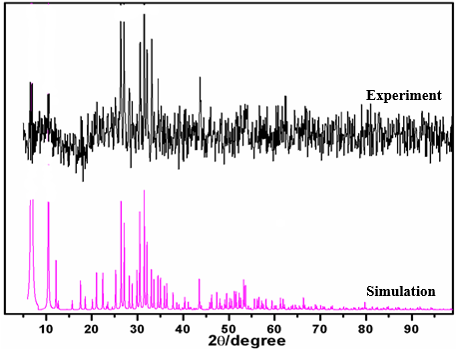


**Figure S3.** The X-ray powder diffraction pattern of compound **1**.


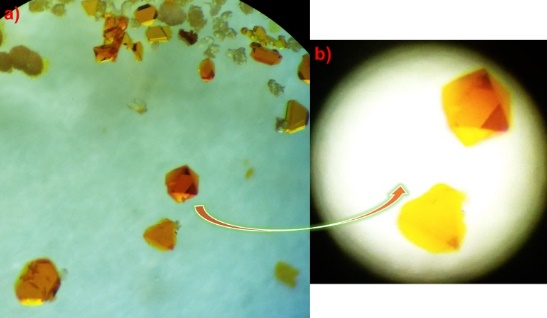


**Figure S4.** Single-crystal morphology observed by optical microscope of compound **1**.


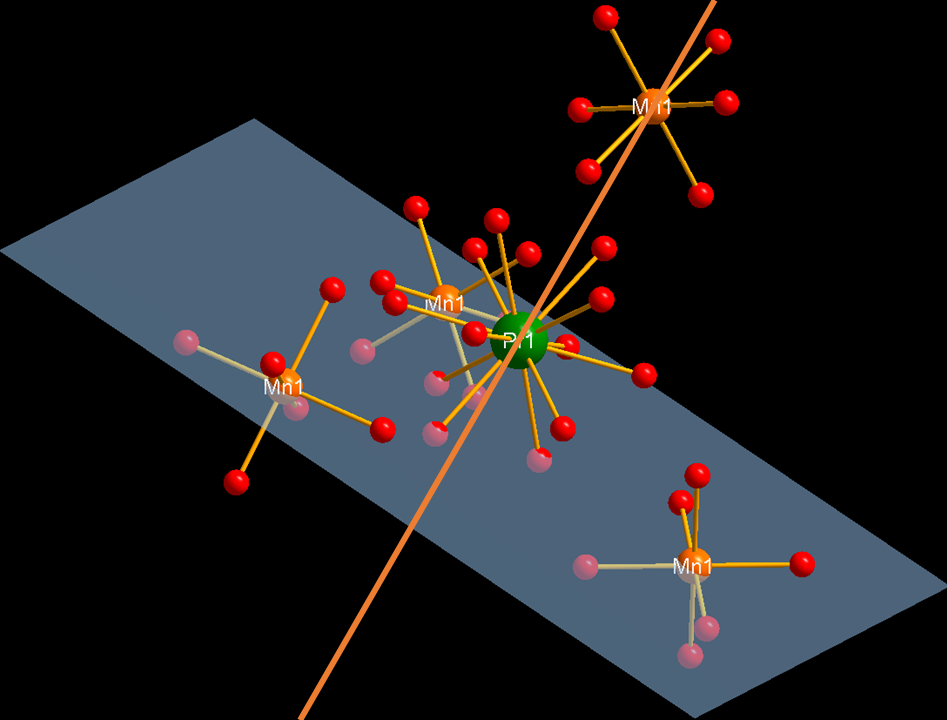


**Figure S5.** The relative position of the Pr^3+^ and Mn^2+^ in compound **1**.


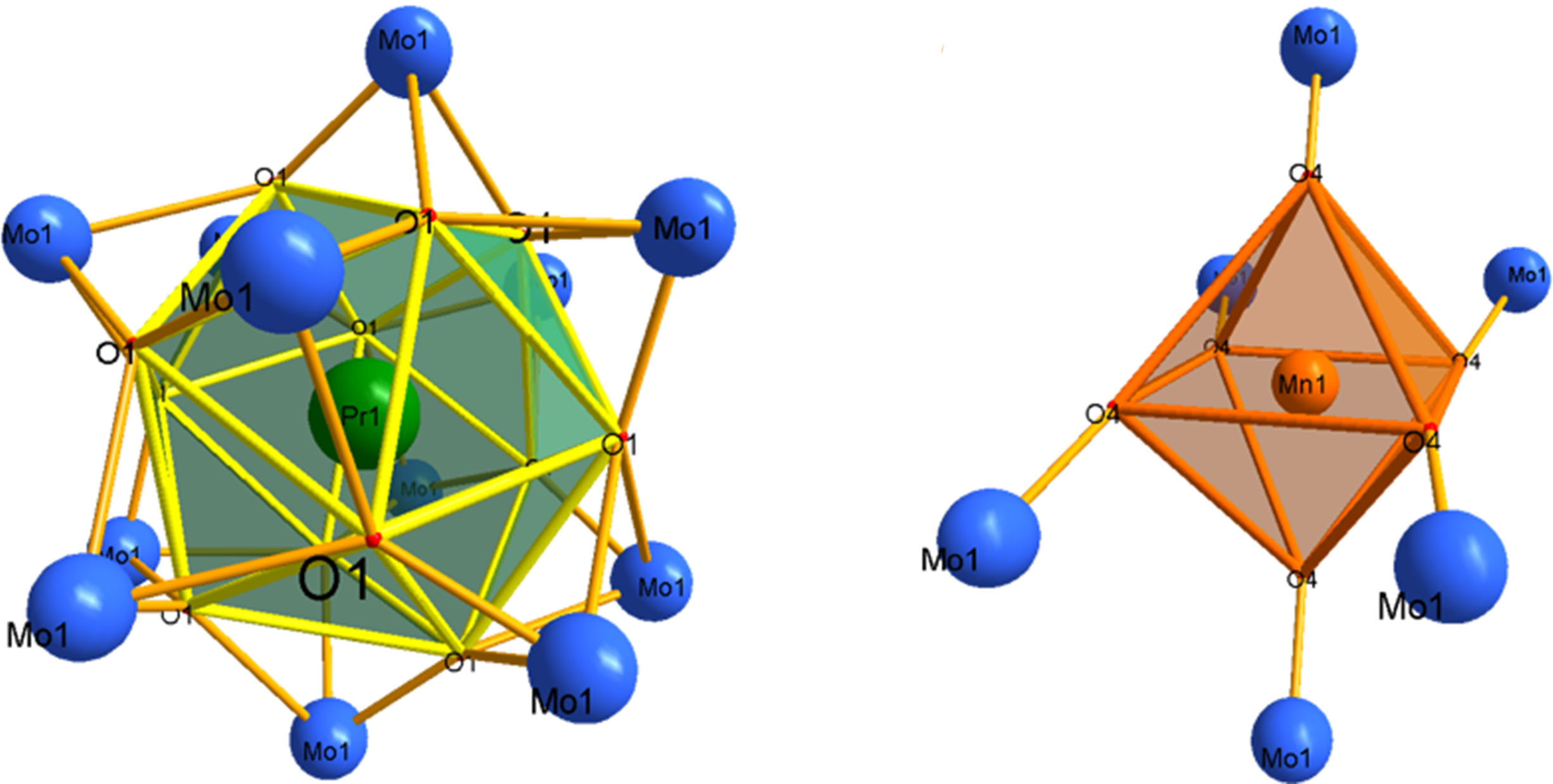


**Figure S6.** The coordination environment of Pr^3+^ and Mn^2+^ in compound **1**.


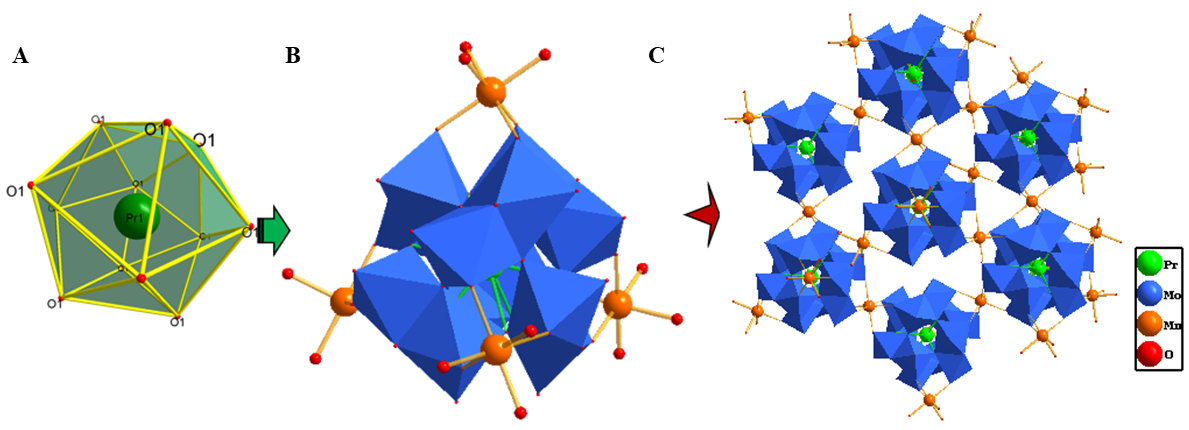


**Figure S7.** The representation of the assembly of compound **1**.


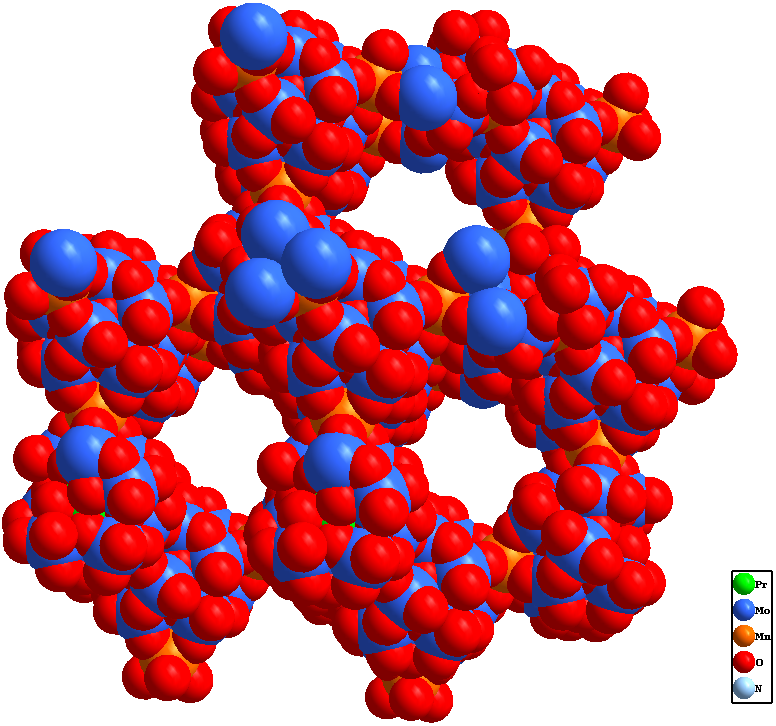


**Figure S8.** The space-filling view of compound **1**.


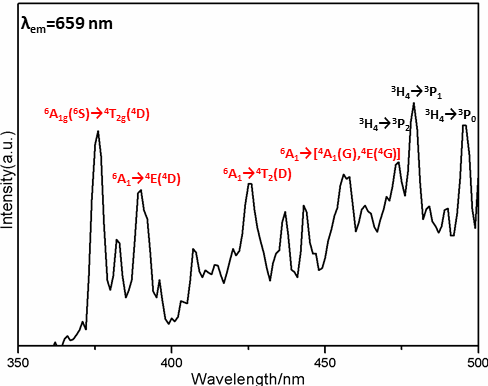


**Figure S9.** The excitation spectrum of compound **1**.


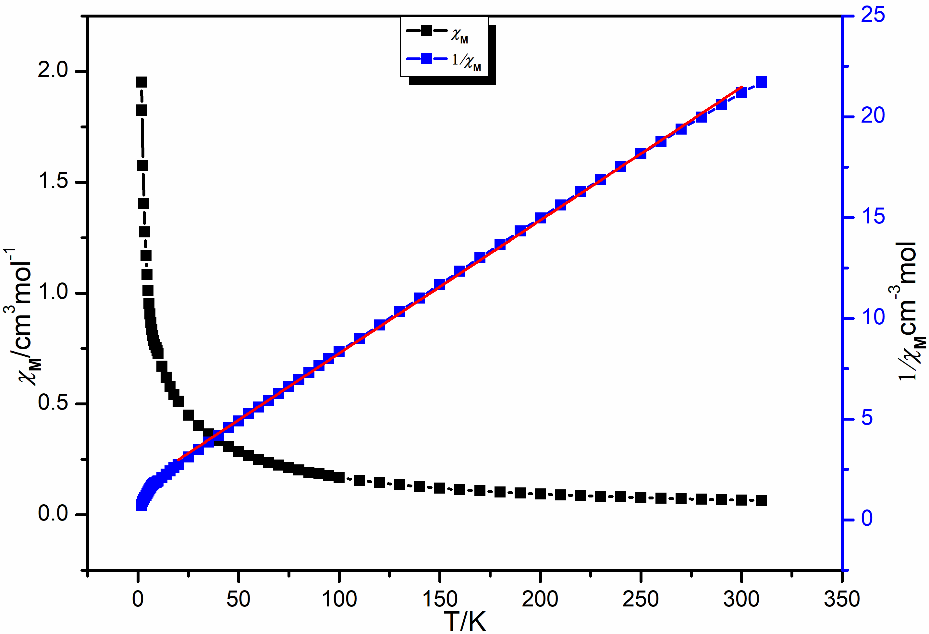


**Figure S10.** Plots of *χ*_M_ and 1/*χ*_M_ *versus* *T* of compound **1**.
